# International collaboration strives for resilient Silk Road at SiDRR Conference 2019

**DOI:** 10.1093/nsr/nwz075

**Published:** 2019-06-18

**Authors:** By Rongzhi Tan, Peng Cui

**Affiliations:** Institute of Mountain Hazards and Environment, Chinese Academy of Sciences, China

Scientists attending the International Conference on Silk-Road Disaster Risk Reduction and Sustainable Development (SiDRR Conference 2019) call for strengthening science and technology innovation and cooperation in disaster prevention and mitigation, and sustainable development to strive for a safe, green and resilient Silk Road.

On 11–12 May 2019, more than 780 experts and scholars from over 40 countries and regions met in Beijing to exchange ideas on how to make the Silk-Road areas more sustainable and disaster-proof. They reached a consensus on disaster risk reduction (DRR) and sustainable development through announcing the Beijing Statement, which is a scientific, technical and political dialogue for better implementation of the Sendai Framework for DRR and Sustainable Development Goals (SDGs) 2030. Aligning with and contributing to the global Science and Technology Road Map, the statement recommended actions on four priorities and nine initiatives, including improving the understanding and management of disaster risk, investing in DRR and SDGs for resilience construction and enhancing disaster preparedness for rapid and effective rescue and reconstruction.

The conference aims to build an updated platform for international coordination in DRR and provide scientific and technological support for the joint construction of the Silk Road and the prosperity of all countries. Under the witness of all participants, the Alliance of International Science Organizations on Disaster Risk Reduction was launched. The alliance has already attracted around 30 organizations and still welcomes new partners to join in. It shares strong interests in DRR and a geographical focus on the Silk-Road regions and the vision of the Sendai Framework, SDGs 2030 and the Paris Agreement on Climate Change.

The alliance emphasizes enhancing disaster mitigation and response through multinational and multidisciplinary cooperation; encouraging partnership with the engineering community; enhancing cooperation between natural and social sciences; focusing on higher education; contributing to disaster-proofing and a resilient infrastructure; and connecting with existing networks as well as private sectors.

The ‘Atlas of Silk Road Disaster Risk’ and the ‘Glance at the Silk Road Disaster Risk’ report will be issued in late 2019. The atlas, with over 120 drawings, will illustrate the physical and social conditions, disaster characteristics and typical disaster events along the Silk Road, visualizing the results of disaster risk assessment at multiple scales, while the report will provide an overall description of risk assessment in Silk-Road areas and share human geographer and social scientists’ experiences in risk governance and management.

Chunli Bai, president of the Chinese Academy of Sciences, said in the opening ceremony that DRR and eco-environmental control are particularly big challenges that we must address collectively in order to build a healthy and resilient Belt and Road and to contribute to the overall global sustainable development. ‘To effectively address these challenges, we have to rely on science and innovations and international cooperation,’ said Bai.

In a video speech, Mami Mizutori, the special representative of the UN Secretary-General for DRR, pointed out that the Belt and Road Initiative (BRI) was one of the most important large-scale infrastructure-development projects being carried out in the world. ‘It is encouraging that the Chinese government is taking proactive measures to ensure the development of BRI risk-informed and sustainable,’ she stressed.

She also emphasized that disaster-proofing the world's largest infrastructure strategy is a challenge, but achievable, since great potential for innovation and creativity lies at the heart of this challenge. Scientists and researchers can use this opportunity to create a new wave of innovation in disaster-resilient infrastructure and wide-scale DRR.

SiDRR Conference 2019 was co-hosted by the Chinese Academy of Sciences, the China Association for Science and Technology, the United Nations Environment Program, the United Nations Office for Disaster Risk Reduction and the Alliance of International Science Organizations.

